# Dispersion, Separation, and Optical Sensing Applications of Single-Walled Carbon Nanotubes

**DOI:** 10.3390/ma19204427

**Published:** 2026-10-21

**Authors:** Zhenyang Huang, Na Lu, Lele Wang, Yanli Wen, Gang Liu, Hongzhen Peng, Lihua Wang

**Affiliations:** 1 School of Materials Science and Engineering, Shanghai University of Engineering Science, Shanghai 201620, China; 17798962373@163.com; 2 Key Laboratory of Bioanalysis and Metrology, State Administration for Market Regulation, Shanghai Institute of Measurement and Testing Technology Co., Ltd., 1500 Zhang Heng Road, Shanghai 201203, China; wangll@simt.com.cn (L.W.); wenyl@simt.com.cn (Y.W.); 3 Institute of Materiobiology, Shanghai University, Shanghai 201800, China; penghongzhen@shu.edu.cn

**Keywords:** single-walled carbon nanotubes, near-infrared, fluorescent, optical biosensing

## Abstract

Single-walled carbon nanotubes (SWCNTs) feature extraordinary electrical, optical and mechanical properties governed by their unique chiral structures, yet as-synthesized SWCNTs exist as heterogeneous mixtures with severe bundling, which severely restricts their high-performance applications. Over recent decades, tremendous progress has been achieved in the dispersion, chiral sorting and optical sensing of SWCNTs. In this review, we first comprehensively summarize the mainstream dispersion strategies of SWCNTs, including physical dispersion relying on external mechanical forces and non-destructive chemical dispersion such as polymer wrapping, DNA wrapping, surfactant adsorption and aromatic small molecule π–π stacking. Subsequently, we systematically introduce several representative chiral sorting techniques, namely aqueous two-phase extraction (ATPE), density gradient ultracentrifugation (DGU), gel column chromatography (GCC), and polymer-based wrapping approach. Furthermore, we highlight the research advances of SWCNT-based near-infrared (NIR) fluorescent sensors for the detection of metal ions, neurotransmitters, nucleic acids, proteins and viruses, as well as their applications in in vivo biological detection of plants and animals. Finally, we discuss the existing challenges in the scalable preparation of chirality-pure SWCNTs, sensor stability and in vivo applications, and prospect the future development directions toward practical translation. This review aims to provide a systematic reference for promoting the development and application of SWCNTs in advanced optical biosensing.

## 1. Introduction

Driven by the burgeoning applications of nanotechnology in fields including electronics [1,2,3], biomedicine [4,5,6,7,8], and catalysis [9,10,11,12,13], carbon nanotubes (CNTs), a unique allotrope of carbon, have drawn extensive research interest since the early 1990s [14,15,16,17,18,19,20,21,22]. Over the past few decades, the application research of CNTs has continuously expanded in scope, largely owing to the exceptional physicochemical properties endowed by the unique chiral structure of single-walled carbon nanotubes (SWCNTs) [23,24,25,26,27]. These properties are intrinsically determined by their chiral structures and can be characterized by chiral indices (*n*,*m*) [28]. However, as-synthesized SWCNTs are typically heterogeneous mixtures comprising multiple coexisting chiralities, mixed metallic and semiconducting species, and bundled aggregates. Such inhomogeneous mixed systems severely limit their practical applications in high-precision electronics, optoelectronics, and optics that demand stringent performance stability and tunability [29,30,31].

To exploit the remarkable functional potential of SWCNTs, researchers have made significant progress in addressing issues relating to SWCNT dispersion, chiral sorting, and optical applications. The strong van der Waals forces between carbon nanotubes [32,33] cause them to aggregate tightly into bundles, making efficient dispersion the primary prerequisite. These bundled structures not only quench the intrinsic near-infrared (NIR) fluorescence of SWCNTs but also impede subsequent purification and practical applications [34,35]. Efficient sorting of SWCNTs by chirality and electronic type is the key to achieving structure-defined properties [36,37], which is particularly crucial for application scenarios that require single-chirality CNTs with uniform bandgaps and optical responses [38]. The (*n*,*m*) chiral indices directly determine the NIR emission wavelength; for instance, (6,5), (7,5), (9,4), and (7,6) species emit at ~990, ~1040, 1115, and ~1130 nm, respectively [39]. Moreover, the fluorescence response to target analytes is highly chirality-specific, with different (*n*,*m*) species exhibiting distinct binding affinities and modulation efficiencies [40,41]. These variations originate from differences in diameter and electronic structure [42,43,44], which govern exciton dynamics, charge transfer rates, and the accessibility of the nanotube surface to analyte molecules. Furthermore, sorted and purified SWCNTs have been successfully applied in optical sensing platforms, leveraging their stable NIR fluorescence, excellent deep tissue penetration, and low background interference [45,46,47].

A number of reviews [24,31,48,49,50,51,52,53,54,55,56,57] have covered advances in SWCNT growth, sorting, and biosensing applications. The intrinsic correlations among these three subfields represent an additional perspective that could be further explored through an integrated framework. However, most of them focus on a single topic. This review lies in integrating dispersion, chiral sorting, and NIR optical sensing into one framework and in emphasizing how chirality purity and dispersion quality influence optical sensing performance. Furthermore, this review highlights recent progress in chirality-pure SWCNT sensors, machine learning-assisted virus identification, and in vivo detection in plants and animals, which are less comprehensively covered in previous reviews. This review is not intended to be a systematic review. Instead, it is a narrative review based on representative studies in the field. The literature was searched mainly through Web of Science using keywords such as “single-walled carbon nanotubes”, “dispersion”, “chiral sorting”, “near-infrared fluorescence”, and “optical sensing”. Studies were selected based on their relevance to SWCNT dispersion, chiral separation, or NIR fluorescent sensing, with preference given to peer-reviewed articles published in recent years. Older references were included when they reported landmark findings. We first elucidate the fundamental principles and mainstream technical strategies for SWCNT dispersion, and provide an overview of various chiral sorting methods along with their landmark research achievements. We then highlight key developments in SWCNT-based NIR fluorescent sensors for diverse detection targets, with an emphasis on sensor design, performance metrics, and practical applications. Finally, we summarize the current bottlenecks in research and present an outlook on future directions in this rapidly evolving field.

## 2. Dispersion of SWCNTs

Efficient dispersion of SWCNTs [31,58] is a prerequisite for their subsequent sorting, functionalization, and device applications. Due to strong van der Waals interactions, as-produced SWCNTs tend to form tightly bundled aggregates [59], which severely compromise their intrinsic properties. This section provides an overview of mainstream SWCNT dispersion strategies, including physical dispersion techniques that rely on external mechanical forces and chemical dispersion methods that stabilize individual nanotubes through covalent or non-covalent interactions [60].

### 2.1. Physical Dispersion Techniques

Physical dispersion techniques are commonly employed to achieve efficient deagglomeration of SWCNTs. These techniques apply external mechanical energy to overcome the agglomeration caused by strong van der Waals forces between nanotubes, while causing no irreversible chemical alteration to the nanotube structure itself.

Among these techniques, ultrasonication has been widely used to disperse SWCNTs in various solvent media and has become one of the most extensively applied physical dispersion methods [61]. Ultrasonication disperses carbon nanotubes by generating transient cavitation effects and microjets in the dispersion medium through high-frequency sound waves, thereby effectively breaking down bundle-like agglomerated nanotubes into individual or small-bundle structures [62,63,64]. Specifically, cavitation bubbles rapidly form, expand, and instantly collapse under ultrasonication. The localized high-energy shockwaves and high-speed microjets released during this process effectively overcome the van der Waals attraction between SWCNTs, disentangling and dispersing the bundled agglomerates, thereby enhancing their dispersibility and stability in liquid media. Cheng et al. [64] further validated this mechanism by systematically investigating the effects of ultrasonication parameters and solvent properties on SWCNT dispersion, confirming the critical role of cavitation effects and microjets in the deagglomeration process. Niyogi et al. [65] studied the interaction between SWCNTs and the solvent o-dichlorobenzene (ODCB) under ultrasonication, elucidating the key factors that stabilize the ultrasonication-based dispersion system. Koshio et al. [66] demonstrated that ultrasonication could induce a chemical reaction between SWCNTs and a solution of poly(methyl methacrylate) (PMMA) in monochlorobenzene (MCB), thereby achieving functionalization of SWCNTs. Furthermore, ultrasonication might damage SWCNTs, controlling ultrasonication parameters is crucial for SWCNT dispersion, as excessive ultrasonication can damage the nanotube structure, leading to the formation of defects and shortened fragments [61,67] which significantly reduce their electrical and mechanical properties. For instance, Pagani et al. [68] revealed that long and short CNTs fracture via buckling and stretching, respectively, which explains the disparate power-law exponents describing CNT length decay under ultrasonication.

In addition to ultrasonication, other physical methods such as mechanical stirring, high-shear homogenization, and ball milling are also commonly used to assist dispersion [69,70,71,72]. Mechanical stirring enables macroscopic mixing, while high-shear treatment further enhances the breakup of agglomerates. These methods are simple to operate, offer controllable conditions, and preserve the intrinsic electrical and optical properties of SWCNTs to the greatest extent. However, because they rely solely on external forces to achieve dispersion [58] without fundamentally altering the surface interactions of the nanotubes, the dispersed nanotubes are prone to re-agglomeration due to inter-tube affinity in the absence of stabilizers such as surfactants, resulting in limited dispersion stability. Therefore, physical dispersion is typically employed as a pretreatment step and is often combined with surfactant addition, polymer modification, or other strategies to obtain stable and uniform SWCNT dispersion systems.

### 2.2. Chemical Dispersion Techniques

Chemical dispersion [73] is one of the most prevalent and widely used strategies for achieving stable dispersion of individual SWCNTs in aqueous or organic solutions. Unlike purely physical dispersion methods, chemical dispersion uses specific chemical interactions to fundamentally overcome the strong inter-tubular van der Waals forces [74]. These chemical interactions act as “barriers” between individual SWCNTs, preventing re-aggregation and enabling the formation of long-term stable dispersions of isolated nanotubes. In general, chemical dispersion approaches can be broadly classified into two major categories based on whether the functional groups are covalently bonded to the SWCNT surface or interact with it through non-covalent forces, namely covalent and non-covalent methods [75]. Covalent functionalization achieves stable surface modification while introducing structural defects, whereas non-covalent functionalization retains the pristine carbon nanotube structure but exhibits weak interfacial binding and a risk of desorption under varying environmental conditions.

Early studies on SWCNT dispersion primarily relied on covalent functionalization, which introduces functional groups to improve hydrophilicity, solubility, and colloidal stability. With the advancement of carbon nanomaterial modification technology, recent advances have focused on defect-engineered SWCNTs [76,77,78], where sp^3^ quantum defects are introduced through covalent modification of the carbon lattice. The mainstream preparation route of this strategy starts with oxidative pretreatment using mixed strong acids (e.g., H_2_SO_4_/HNO_3_) [79]. This oxidative treatment selectively acts on the inherent structural defect sites of SWCNTs, generating abundant carboxylate active groups on the nanotube sidewalls. These moieties are then exploited via nucleophilic substitution reactions (e.g., amidation or esterification) [80,81] to attach bulky alkyl chains or hydrophilic polymers onto the sidewalls. Advanced chemical methods, including the Billups–Birch reduction [82,83] and 1,3-dipolar cycloaddition [84,85], further broaden this repertoire by grafting aryl or alkyl groups directly onto the carbon lattice. Covalent functionalization via quantum defects retains the desirable NIR photoluminescence of SWCNTs, allowing practical applications including dopamine and serotonin sensing [86,87], insulin detection [88], pyrophosphate sensing [89], and bioimaging [90,91].

Non-covalent functionalization [58] provides a mild, non-destructive strategy that allows for efficient and uniform dispersion of SWCNTs while preserving their intrinsic sp^2^ carbon framework and exceptional properties. This approach primarily relies on weak supramolecular interactions, including π-π stacking, hydrophobic effects, and electrostatic adsorption [57], to stably anchor functional moieties onto the SWCNT sidewalls, effectively suppressing van der Waals aggregation among nanotubes. Representative dispersion strategies include polymer wrapping, DNA wrapping, surfactant-mediated interfacial adsorption, and aromatic small molecules-induced π-π stacking dispersion [57].

#### 2.2.1. Polymer Wrapping

Conjugated polymer wrapping [92,93] is one of the most widely adopted strategies for the non-covalent dispersion of SWCNTs [94]. The underlying mechanism involves the formation of strong π-π stacking interactions between the π-conjugated system of the polymer chains and the sp^2^ carbon framework on the SWCNT sidewalls, while the steric hindrance provided by the polymer chains effectively suppresses van der Waals-induced aggregation among SWCNTs, thereby enabling their uniform dispersion in solvents. In this strategy, the structural compatibility between the conjugated polymer and the SWCNT surface is a critical determinant of dispersion efficiency. Commonly used conjugated polymers include polyfluorene (PFO) [95,96], polythiophene (P3AT) [97], poly[(9,9-dioctylfluorene)-alt-(1,4-benzo-2,10,3-thiadiazole) (F8BT) [95,98], poly-[9,9-di(N,N-dimethylaminopropyl)fluorene] (PFDMA) and poly[9,9-di(N,N,N-trimethylammoniumpropyl)fluorene dibromide] (PFAB) [99].

**Figure 1:**
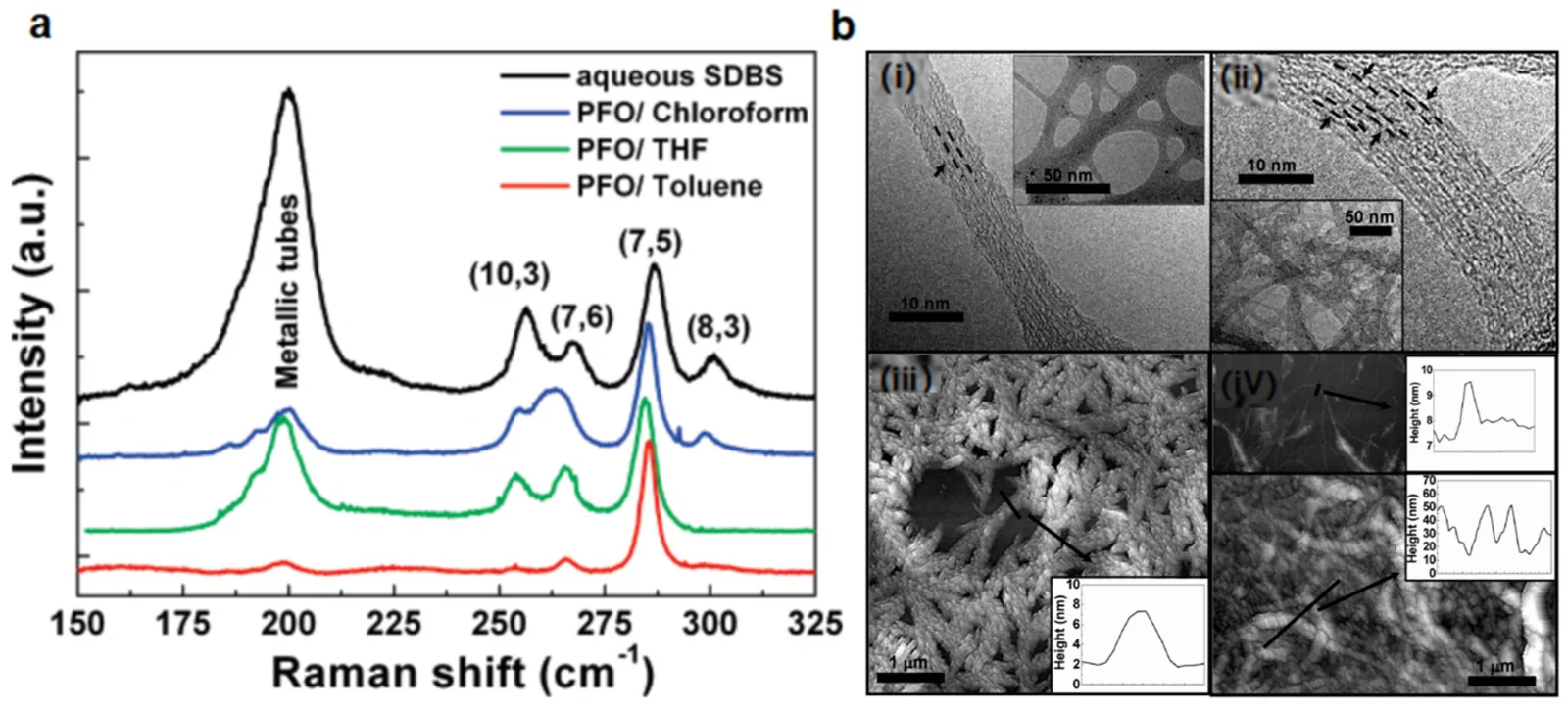
(**a**) Raman spectra of SWCNTs dispersed in SDBS aqueous solution and in PFO/organic solvents, excited at 647 nm by a Kr^+^ laser. (**b**) Transmission electron microscopy (TEM) and atomic force microscopy (AFM) images of SWCNTs dispersed using PFO in toluene (**i**,**iii**) and chloroform (**ii**,**iv**). Adapted with permission from Ref. [100],© American Chemical Society 2008 and 2011.

In 2007, Nish et al. [95] first used PFO and F8BT to selectively dispersion of semiconducting SWCNTs. The results showed that this method exhibited a higher selectivity for sorting semiconducting SWCNTs than methods using bile acid salts. Hwang et al. [100] investigated the effects of different polymer structures and organic solvents on the selectivity of SWCNT sorting (Figure 1). They found that the highest selectivity was observed when using toluene as the solvent together with the rigid polymers PFO-BT and PFO to suspend and separate the nanotubes. When chloroform was used as the solvent, most of the dispersed carbon nanotubes existed as bundles, and the sorting selectivity decreased. Other polyfluorene-based copolymers were less efficient than PFO in differentiating semiconducting SWCNTs.

Beyond conjugated polymers, polyethylene (PEG)-based polymers and PEG-lipid functionalization strategies are prevalent non-covalent strategies for aqueous SWCNT stabilization suitable for biomedical applications [101,102,103]. Different from the π-π stacking mechanism of conjugated polymers, lipid-PEG anchors to SWCNT sidewalls via hydrophobic interactions, while the outer hydrophilic PEG chains form a dense hydration layer. Dai et al. first used phospholipid-PEG glycol as the key dispersant for SWCNTs, replacing sodium cholate via a two-step surfactant exchange to preserve bright NIR fluorescence while imparting excellent biocompatibility for in vivo imaging in mice [101]. Kalinina et al. reported the use of tetrahydrofurfuryl-terminated polyethylene glycol (PEG-THFF, MW∼200) to covalent functionalized SWCNTs via sequential ultrasonication and high-shear mixing at two distinct length scales [104].

#### 2.2.2. Biomolecule Wrapping

Biomolecule wrapping dispersion strategy utilizes the unique structures of biomolecules such as DNA, peptides, and polysaccharides to bind to SWCNTs through multiple weak interactions (e.g., π-π stacking, hydrophobic effects, hydrogen bonding, and electrostatic adsorption), forming a bio-coating layer. This approach suppresses nanotube aggregation via steric hindrance while exploiting the hydrophilicity of the biomolecules to achieve stable dispersion of SWCNTs in aqueous or physiological environments. Representative biomolecules include single-stranded DNA (ssDNA) [105,106,107,108,109], poly-L-lysine (PLL) [110], and chitosan (CS) [111,112,113]. ssDNA adsorbs onto the SWCNT sidewalls through π-π stacking interactions between its nucleobases and the nanotube surface [106,114], while its hydrophilic phosphate backbone enables uniform dispersion in aqueous solutions. Moreover, the dispersion selectivity toward specific SWCNT diameters can be tuned by adjusting the DNA sequence [115]. PLL, a cationic polypeptide, binds to the weakly negatively charged SWCNT surface via electrostatic interactions; the steric hindrance and hydrophilicity provided by its molecular chains facilitate effective nanotube dispersion, and the material exhibits excellent biocompatibility. Chitosan, a natural polysaccharide, forms hydrogen bonds and electrostatic interactions with SWCNTs through its hydroxyl and amino groups, achieving wrapping and dispersion of the nanotubes. Its biodegradability renders it particularly suitable for SWCNT dispersion applications in the biomedical field.

**Figure 2:**
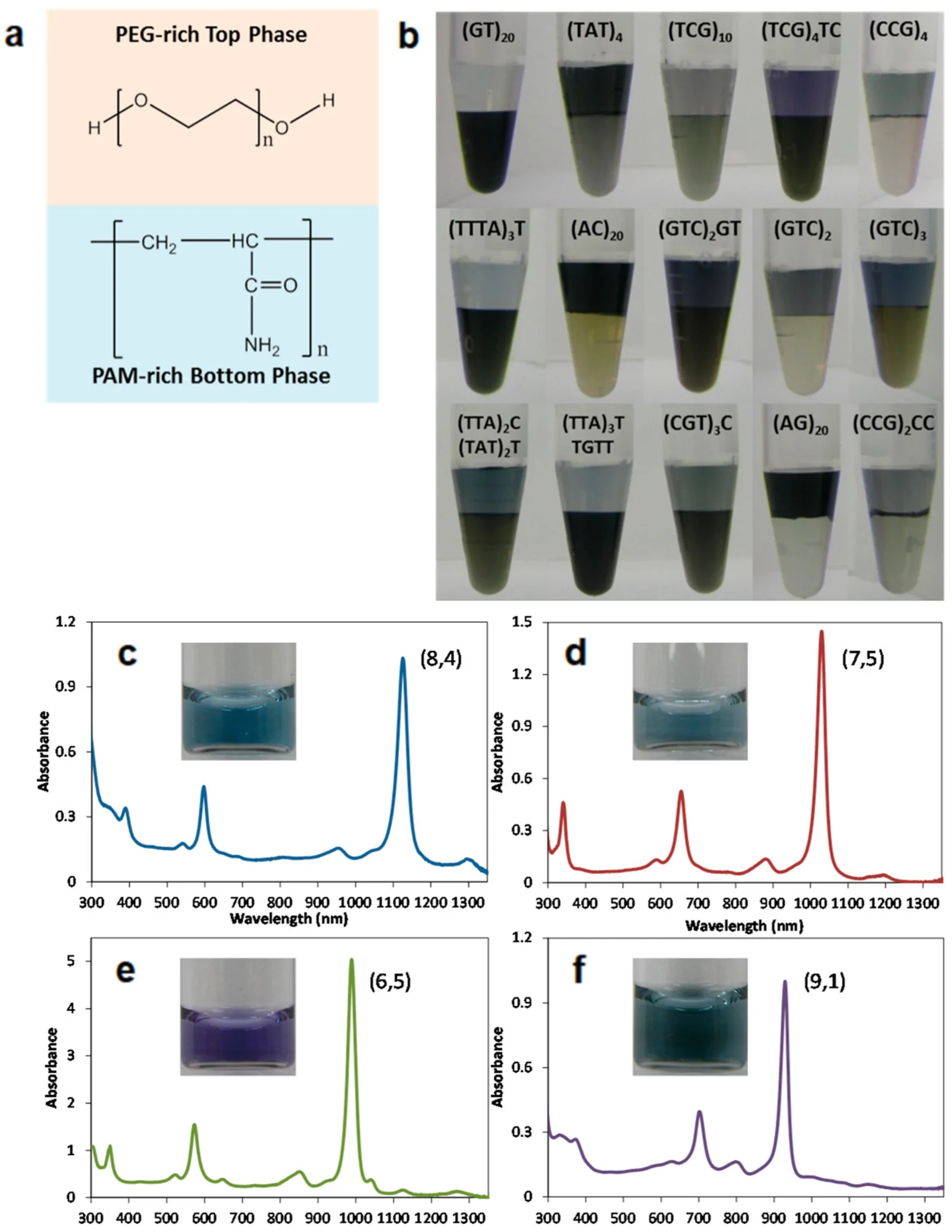
Sequence-dependent partition of DNA-SWCNTs in the PEG/PAM two-phase system. (**a**) Schematic diagram of the PEG/PAM system. (**b**) Photographs showing the partition of various DNA-SWCNTs in the PEG/PAM system. (**c**–**f**) UV-vis-NIR absorption spectra of highly enriched (*n*,*m*) CNT species: (**c**) (8,4) from (GT)20-SWCNTs; (**d**) (7,5) from (TATT)3T-SWCNTs; (**e**) (6,5) from (TAT)4-SWCNTs; (**f**) (9,1) from (GTC)2GT-SWCNTs. Adapted with permission from Ref. [107], © American Chemical Society 2014.

DNA wrapping plays a crucial role in the dispersion of SWCNTs. In 2003, Zheng et al. [106] first achieved the dispersion of bundled SWCNTs in aqueous solution by sonication in the presence of ssDNA. The hydrogen-bonded periodic purine-pyrimidine two-dimensional sheets are capable of selectively wrapping specific nanotubes. Moreover, they [107] systematically investigated the spontaneous partition behavior of DNA-wrapped SWCNTs in polymer aqueous two-phase systems. The results (Figure 2) revealed that the partition of DNA-wrapped SWCNTs exhibited a clear sequence-dependent manner, and could be further regulated by salt and polymer additives. By rationally combining DNA sequences, polymer aqueous two-phase systems, and partition modulators, up to 15 single-chirality nanotube species could be effectively purified. Liu et al. [95,116] dispersed SWCNT powder in the presence of DNA under ice-bath conditions by sonication, then removed impurities by centrifugation and collected the resulting DNA-SWCNTs dispersion.

#### 2.2.3. Surfactant Adsorption

The interfacial adsorption strategy employing surfactants and amphiphilic block copolymers relies on the amphiphilic Nature of these molecules. Specifically, hydrophobic moieties (e.g., alkyl chains, aromatic rings) adsorb onto the hydrophobic sidewalls of SWCNTs via hydrophobic interactions, while hydrophilic groups (e.g., carboxyl, sulfonate, polyethylene glycol chains) form hydrogen bonds with water or polar solvents. This reduces the surface energy of SWCNTs in polar media, disrupts inter-tube aggregation, and achieves debundling and dispersion. Typical dispersing agents include the anionic surfactant sodium dodecyl sulfate (SDS) and sodium dodecylbenzenesulfonate (SDBS) [117], the cationic surfactant cetyltrimethylammonium bromide (CTAB), and the amphiphilic block copolymer polyethylene glycol-polypropylene glycol-polyethylene glycol (PEG-PPG-PEG, F127) [118].

For anionic surfactants like SDS [34] and SDBS [119], the hydrophobic alkyl chain adsorbs onto the SWCNT surface, while the hydrophilic head group (sulfate for SDS, sulfonate for SDBS) imparts a negative charge, promoting dispersion through electrostatic repulsion. Smalley et al. [34] sonicated an aqueous dispersion of SWCNTs in SDS, followed by centrifugation to remove bundles, ropes, and residual catalysts, obtaining individual nanotubes. Yodh et al. [120] reported the solubilization of high-weight-fraction SWCNTs in water using SDBS. Atomic force microscopy measurements showed that even at a high concentration of 20 mg/mL, approximately 63 ± 5% of the bundles were effectively exfoliated into individual nanotubes. In contrast, CTAB relies on electrostatic adsorption between its cationic head and the weakly negatively charged SWCNT surface, with additional steric hindrance from its hydrophobic tail further enhancing dispersion. F127, with its hydrophobic PPG block adsorbing onto the nanotube surface and its hydrophilic PEG blocks extending into the aqueous phase, enables high-concentration, stable dispersion of SWCNTs in aqueous solutions.

#### 2.2.4. π-π Stacking with Aromatic Small Molecules

π-π stacking dispersion strategy using polycyclic aromatic hydrocarbons (PAHs) exploits the large π-conjugated system of PAH molecules (e.g., pyrene derivatives) to form strong π-π stacking interactions with the sp^2^ carbon framework on the SWCNT sidewalls [121,122]. Concurrently, the hydrophilic or solvophilic groups appended to the PAH molecules improve the compatibility of SWCNTs with the solvent, thereby suppressing nanotube aggregation and achieving efficient dispersion. Commonly employed PAHs include 1-pyrenebutyric acid (PBA) [123], pyrenecarboxaldehyde (PA) [124], and pyrene derivatives [125]. In PBA, the pyrene ring adsorbs onto the SWCNT surface via π-π stacking, while the carboxyl group imparts hydrophilicity to the nanotubes, enabling their dispersion in aqueous solutions. PA utilizes stable π-π interactions between its pyrene ring and the carbon nanotubes, with the aldehyde group providing a site for subsequent functionalization while also facilitating dispersion in polar solvents. Py derivatives combine the π-π stacking capability of the pyrene ring with the hydrophilicity and steric hindrance of PEG chains, achieving both efficient SWCNT dispersion and enhanced biocompatibility and water solubility.

Table 1 compares four non-covalent SWCNT dispersion strategies with different intermolecular interactions. Polymer wrapping achieves high selectivity and stable aqueous dispersion but is limited by solvent dependence, high cost and poor industrial scalability. DNA/ssDNA wrapping offers biocompatible chiral sorting, yet suffers from instability, environmental sensitivity, high cost and slow processing. Surfactant adsorption is simple, low-cost and scalable, but lacks chiral selectivity and encounters removal and cytotoxicity issues. Aromatic small molecules preserve nanotube structures cheaply, constrained by poor dispersion and time-dependent desorption.

Although Table 1 summarizes the main non-covalent dispersion strategies, a more critical comparison is still needed. In terms of stability, polymer wrapping and DNA wrapping generally provide more stable dispersions than surfactant adsorption and aromatic small molecules, because the wrapping layers suppress re-aggregation more effectively. In terms of efficiency, surfactant adsorption is simple and low-cost, but its chirality selectivity is limited. Polymer wrapping can offer high selectivity for certain (*n*,*m*) species, yet it often depends on specific solvents and requires removal of excess polymer. DNA wrapping provides sequence-dependent selectivity and good aqueous dispersibility, but the high cost and limited thermal stability restrict its scalable use. Aromatic small molecules preserve the SWCNT structure and allow further functionalization, but their dispersion stability is often insufficient. Therefore, no single dispersion method simultaneously satisfies high stability, high efficiency, low cost, and scalability; the choice should be made according to the target application.

## 3. Sorting of Chiral SWCNTs

The electrical, optical, and optoelectronic properties of chiral SWCNTs are strictly determined by their chiral structures *(n*,*m*). However, as-prepared products are typically mixtures of multiple chiralities, which fail to meet the requirements for high-precision device applications. Therefore, achieving efficient, high-purity separation of chiral SWCNTs is the key to advancing them from laboratory research toward industrial applications. Current mainstream separation methods for SWCNTs mainly include aqueous two-phase extraction (ATPE), density gradient ultracentrifugation (DGU), gel column chromatography (GCC), and polymer-based wrapping sorting, which achieve high-purity separation based on subtle differences in density, partition coefficients, and gel adsorption affinity among different chiral carbon nanotubes.

### 3.1. Aqueous Two-Phase Extraction (ATPE)

ATPE [126] is a mild separation method that achieves separation based on differences in the partition coefficients of different chiral SWCNTs between two aqueous phases, and has become one of the important techniques for the separation of chiral SWCNTs. In this method, two water-soluble polymers (e.g., polyethylene glycol/dextran) or a polymer and a salt (e.g., polyethylene glycol/potassium phosphate) are mixed, and under appropriate conditions of concentration, temperature, and pH, two immiscible aqueous phases spontaneously form. These two phases exhibit significant differences in interfacial tension, hydrophobic interactions, and electrostatic effects, providing a thermodynamic basis for chiral-selective partitioning. After being wrapped by surfactants (e.g., SDBS, DOC) or biomacromolecules (e.g., single-stranded DNA), SWCNTs with different *(n*,*m*) chiral configurations show subtle differences in surface charge, hydrophobicity, and spatial conformation. As a result, they interact differently with the two-phase system, leading to significant differences in their partition coefficients. Through multistage extraction operations, stepwise separation and enrichment of different chiral SWCNTs can be achieved.

**Figure 3:**
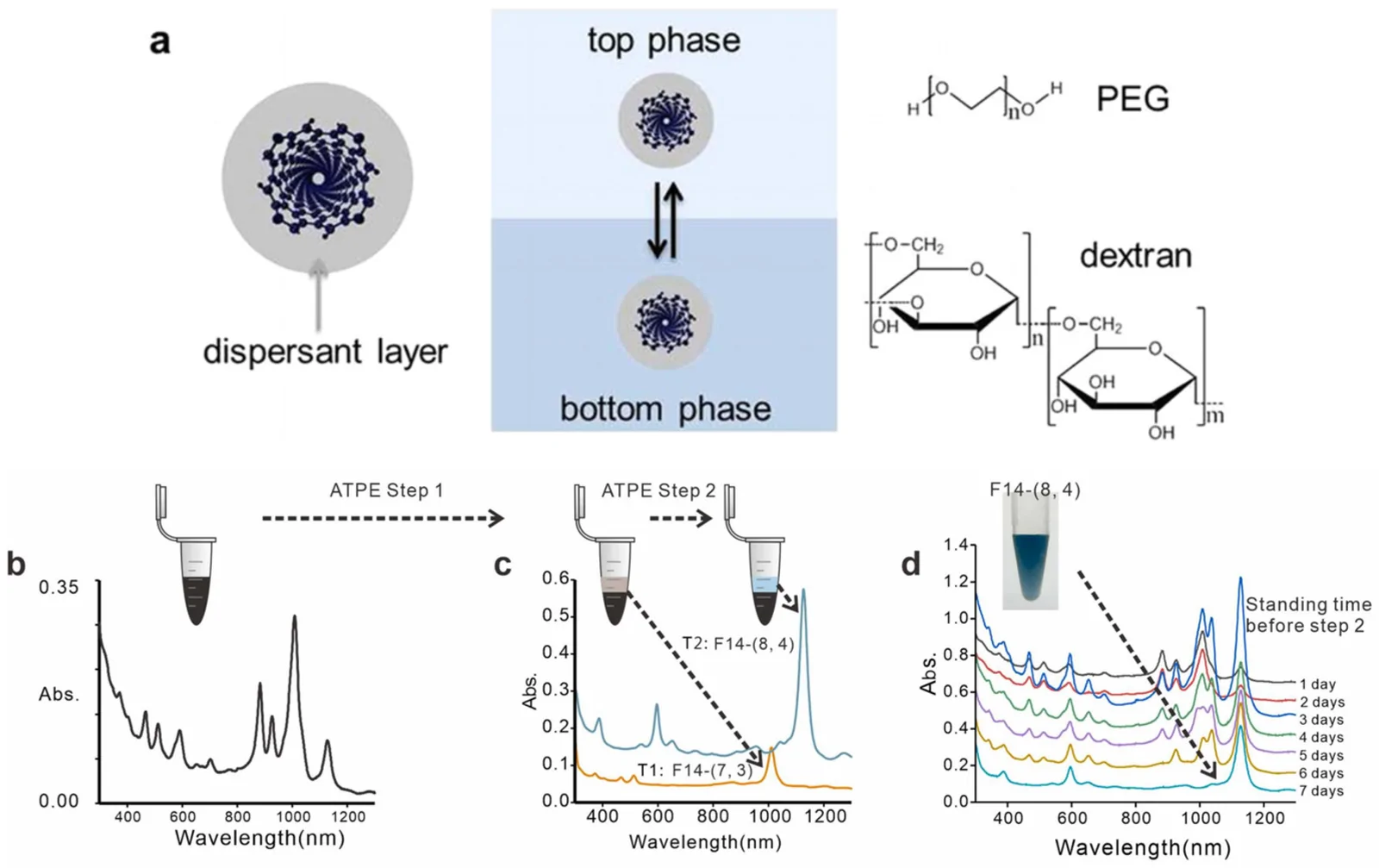
(**a**) Schematic diagram of spontaneous stratification of carbon nanotubes in a PEG/dextran two-phase system [127] (**b**,**c**) UV-Vis-NIR absorption spectra of multi-chirality CNTs (**b**), and single-chirality (8,4) in the T2 phase and (7,3) in the T1 phase (**c**). (**d**) Purity and dispersity of the second top phase (T2) extracted after different standing times following the first step. (**a**) Adapted with permission from Ref. [127], © American Chemical Society 2013. (**b**–**d**) Adapted with permission from Ref. [108], © Elsevier 2025.

Zheng and coworkers pioneered uncovered the sequence-dependent interaction between ssDNA and SWCNTs [106]. The d(GT)_n_ sequences (*n* = 10–45) can helically wrap SWCNTs, tune the electrostatic properties of the resulting hybrids, and achieve separation of nanotubes by metallic/semiconducting type and diameter via anion-exchange chromatography. Then, they first proposed the ATPE strategy [127], using PEG and dextran to form two immiscible aqueous phases. In this PEG/dextran two-phase system, carbon nanotubes spontaneously partition based on their diameter and metallicity (Figure 3). Fagan et al. [128] further optimized the separation conditions by varying the combination of nanotube dispersants, achieving continuous and rapid separation of multiple individual small-diameter SWCNT species with extremely high purity. Li et al. [129] developed a pH-driven ATPE strategy requiring only one to three simple steps, through which they successfully isolated 11 distinct (*n*,*m*) species of SWCNTs with diameters ranging from 0.69 to 0.91 nm. Very recently, Liu’s group [108,116] employed a sorting DNA in a PEG/dextran system to enrich (7,3) single-chirality carbon nanotubes from multi-chirality CNTs (Figure 3a) in the first top phase (T1). By optimizing the extraction system, they obtained high-purity (8,4) CNTs from the second top phase (T2) (Figure 3b). As shown in Figure 1b(iii), the purity of (8,4) CNTs in T2 gradually increased with extended standing time, while non-(8,4) CNTs slowly migrated downward to the bottom phase. In comparison with the multi-chirality CNTs, the purified (8,4) CNTs showed narrow UV-vis-NIR absorption peaks, and the purity of (8,4) was determined to be 96%.

### 3.2. Density Gradient Ultracentrifugation (DGU)

DGU [130] is a high-precision sorting method based on density differences among chiral SWCNTs. Due to subtle differences in the buoyant density of SWCNTs regulated by their surface coatings (surfactants or polymers), different chiralities migrate to their respective buoyant equilibrium positions under ultrahigh centrifugal forces (≥100,000 rpm), forming distinct enrichment bands within the density gradient. By collecting these bands layer by layer, followed by dialysis and centrifugal purification, high-purity single-chirality SWCNTs can be obtained. In 2006, Hersam’s group [131,132] first used DGU to separate DNA- or surfactant-wrapped SWCNTs in an aqueous phase. Under high centrifugal force, differences in the buoyant density, which are determined by the diameter and electronic type of the SWCNTs, lead to the formation of distinct bands in the density gradient, enabling size- and conductivity-selective sorting of carbon nanotubes. In addition, DGU method not only enabled the separation and enrichment of left- and right-handed SWCNT enantiomers using chiral surfactants but also allows for the effective separation of SWCNTs of different chiralities [133] through a dual-surfactant system [134]. More importantly, this method is also applicable to the enrichment of armchair carbon nanotubes [135].

### 3.3. Gel Column Chromatography (GCC)

GCC [136] is currently one of the most widely used methods for the laboratory-scale preparation of chiral SWCNTs. In this method, a porous gel (e.g., Sephacryl, Sepharose) serves as the stationary phase, while a buffer solution supplemented with surfactants acts as the mobile phase. After being coated with surfactants, different chiralities of SWCNTs exhibit variations in the conformational structure of the surfactant shell on their surfaces, leading to distinct selective adsorption affinities toward the gel matrix. These differences in adsorption affinity result in varying permeabilities within the gel pores, thereby enabling the separation of SWCNTs by chirality.

Kataura et al. [137] first reported the selective adsorption effect of SWCNTs on agarose gel, and established a rapid and scalable method to separate metallic and semiconducting SWCNTs. High-efficiency separation of the two types of nanotubes can be achieved by sequentially freezing, thawing and squeezing the gel loaded with SWCNTs and SDS. With this technique, metallic SWCNTs with a purity of 70% are collected in the supernatant, while semiconducting SWCNTs with a purity up to 95% are retained in the gel phase. In 2011, Liu and colleagues [136] established a simple single-surfactant multi-column gel chromatography separation system that enabled the large-scale separation of single-chirality SWCNTs. This technique is based on the differential interactions between surfactant-stabilized dispersed SWCNTs and allyl dextran gel, in which nanotubes with stronger binding affinity to the gel exhibit longer retention times, while those with weaker binding affinity are eluted first, thereby allowing the separation of 13 different chiral carbon nanotube species. Further, the Kataura group [138] optimized the GCC technique by exploiting the difference in interactions between the two isomers of chiral SWCNTs and the allyl dextran-based gel. They successfully separated nine chiral enantiomer pairs, making this one of the methods capable of resolving the greatest number of chiral carbon nanotube species to date.

### 3.4. Polymer-Based Wrapping Sorting

Polymer-based single-chirality enrichment has emerged as a powerful strategy for scalable preparation of monochiral SWCNTs. This approach relies on conjugated polymers such as PFO and its derivatives, which selectively wrap specific (*n*,*m*) chiralities through π-π interactions, thereby enabling direct dispersion of narrow-chirality populations in organic solvents. Kruss et al. [139] used poly[(9,9-dioctylfluor-enyl-2,7-diyl)-alt-(6,6′)-(2,2′-bipyridine)] (PFO-BPy) to enrich (6,5) SWCNTs, and this method was also applicable to other PFO derivatives for the enrichment and functionalization of (7,5) SWCNTs. Zaumseil and co-workers [140] investigated PFO-BPy-sorted single-chirality small-diameter (6,5) and mixed large-diameter SWCNT networks with varying densities and length distributions. Janas et al. [141] utilized a cyclic conjugated polymer extraction (CPE) in combination with a new designed CP, improving the sorting proportions of (6,5) and (7,5) SWCNTs to 25% and 10%, respectively.

Table 2 summarizes the core principles, strengths, limitations and representative outcomes of ATPE, DGU, GCC and polymer-based wrapping sorting for chiral SWCNT separation. All these techniques exhibit inherent trade-offs among purity, yield, processing time and scalability.

## 4. Applications of SWCNTs in Optical Sensing

Recent advances in NIR fluorescence based on SWCNTs have provided new opportunities for optical sensing applications. By integrating strategies such as DNA sequence design, peptide corona functionalization, and chirality control, researchers have successfully established highly sensitive and selective recognition systems for environmental monitoring, disease biomarker detection, and in vivo applications. This section reviews key research progress in this field, with an emphasis on sensing applications targeting various analytes, including metal ions, neurotransmitters, nucleic acids, proteins, viruses, and in vivo detection.

### 4.1. Metal Ions

**Figure 4:**
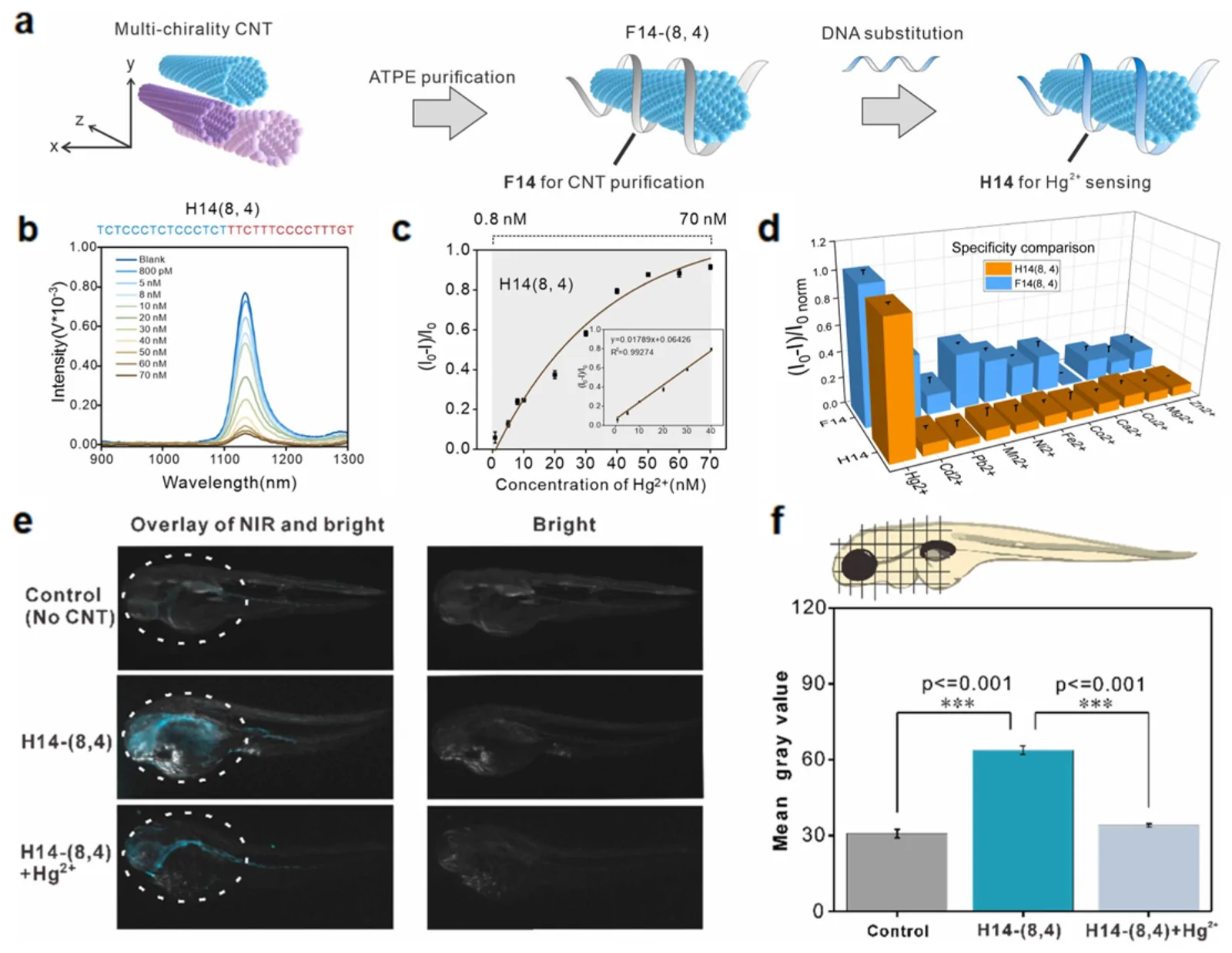
High-selectivity NIR biosensor for Hg^2+^ detection based on a DNA-wrapped (8,4) monochiral carbon nanotube. (**a**) Schematic illustration of sensor construction. (**b**,**c**) Sensitivity response curve. (**d**) Selectivity assay. (**e**,**f**) NIR imaging after the addition of Hg^2+^ in zebrafish. Adapted with permission from Ref. [108], © Elsevier 2025.

In recent years, NIR fluorescence sensing platforms based on SWCNTs have achieved significant progress in the field of metal ion detection. Through strategies such as DNA sequence wrapping, peptide corona functionalization, and chirality control, researchers have successfully constructed a variety of highly sensitive and selective ion recognition systems and explored their practical applications in environmental monitoring [108,116,142,143,144]. Liu’s group [108] employed high-purity (96%) the (8,4) chirality carbon nanotubes and wrapped them with a designed thymine-rich DNA on their surface to construct a NIR sensing platform for the detection of Hg^2+^ (Figure 4). Based on the affinity and interaction between T-Hg^2+^, the helical structure of the DNA-carbon nanotube was affected, thereby altering its fluorescence emission properties. This DNA-carbon nanotube NIR sensor exhibited a good linear response for Hg^2+^ detection in the range of 0.8 nM to 40 nM, with a detection limit of 0.472 nM. Moreover, the sensor demonstrated excellent selectivity against ten common metal ions, enabled reliable quantitative detection of Hg^2+^ in complex river water samples, and successfully achieved real-time in vivo imaging in zebrafish. In addition, this research group [116] combined cytosine-rich DNA sequences with purified the (8,4) chirality carbon nanotubes, leveraging the Ag^+^-C interaction and its resulting NIR response, successfully constructed a fast-response (<1 min) and highly selective Ag^+^ sensing platform. When applied to real water samples, this sensor achieved a good linear detection range for Ag^+^ from 30 nM to 60 μM and exhibited satisfactory recoveries (97.2–107.6%) and favorable precision (RSD of 1.7–6.3%).

Unlike the aforementioned works that focused on the detection of specific ions, Strano et al. [144] systematically investigated the interactions between 35 DNA-wrapped helical structures and 9 dilute divalent metal cations. The results showed that SWCNTs with DNA-wrapped helical structures typically exhibited two distinct sensing states at pH 6 and pH 8, enabling effective differentiation among Hg^2+^, Pb^2+^, Cr^2+^, and Mn^2+^. Furthermore, nanomolar-level sensitive detection of Hg^2+^ in fish tissue extracts was achieved.

In addition to the DNA wrapping strategy, peptide corona functionalization represents another important class of SWCNT modification methods. Bisker et al. [143] employed an enzymatic oxidative polymerization method to polymerize a fluorenylmethyloxycarbonyl-protected tyrosine (FmocY) precursor into a melanin-like substance, which was then used to functionalize single-walled carbon nanotubes. Upon binding with a panel of primarily divalent transition metal ions, including Mg^2+^ and Ca^2+^, the fluorescence emission of the functionalized single-walled carbon nanotubes was modulated, exhibiting a concentration-dependent response with a detection limit in the nanomolar range. Subsequently, this research group [142] developed a sensor library based on peptide corona-functionalized SWCNTs. Combined with an analyte classification and feature selection algorithm, they achieved the identification of transition metal ions Cu^2+^, Ni^2+^, Cr^3+^, Pb^2+^, and Hg^2+^ through fluorescence response detection.

### 4.2. Neurotransmitters

**Figure 5:**
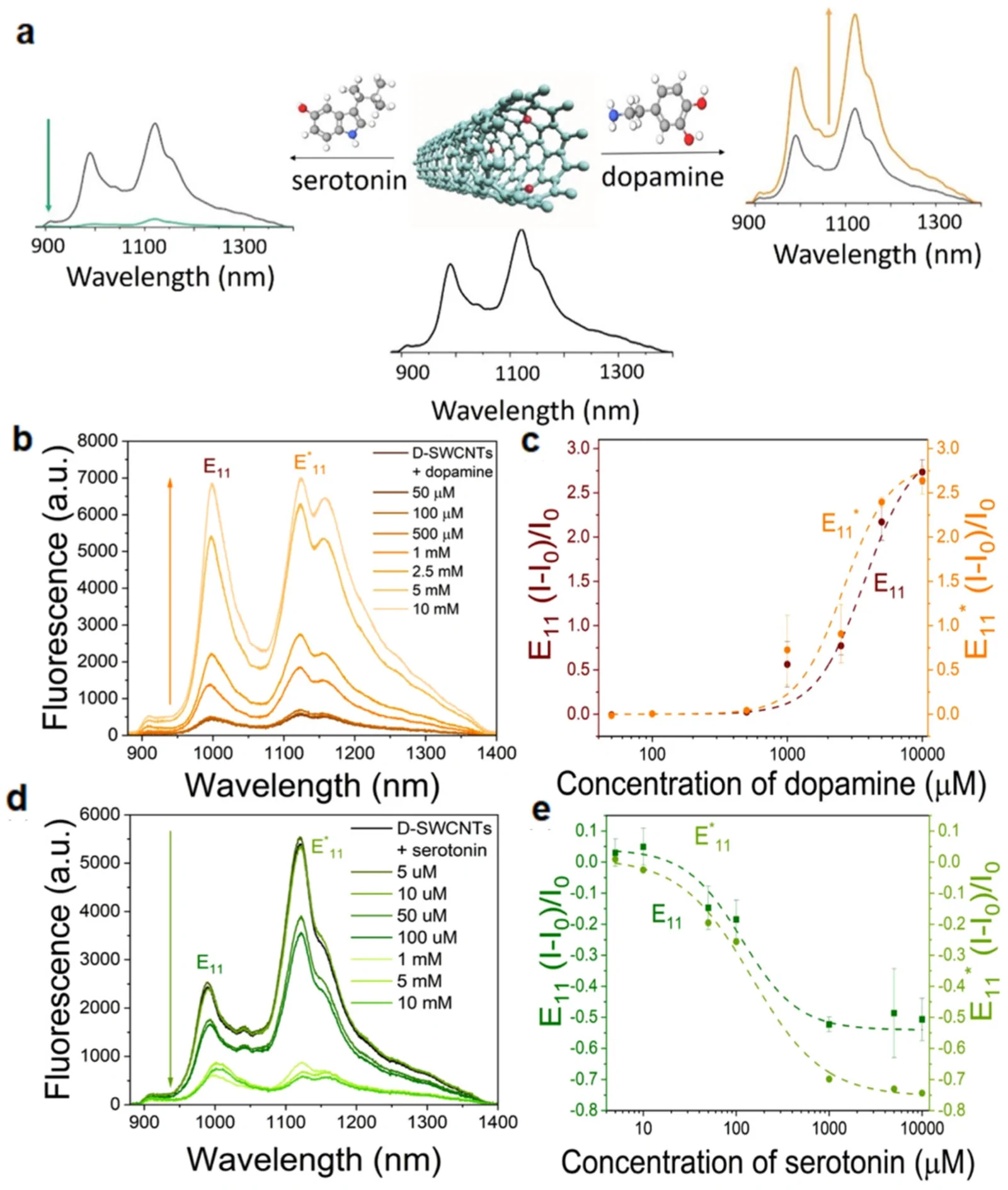
(**a**) Oxygen-defective (6,5) chiral D-SWCNTs and the fluorescence enhancement effect of dopamine and the fluorescence quenching effect of serotonin on these nanotubes. (**b**,**c**) Fluorescence spectra of D-SWCNTs upon addition of different concentrations of dopamine (**b**) and serotonin (**c**). (**d**,**e**) Fluorescence response curves of dopamine (**d**) and serotonin (**e**). Adapted with permission from Ref. [86], © American Chemical Society 2024.

Beyond metal ions, SWCNTs have recently emerged as promising NIR fluorescent probes for the sensitive detection of small-molecule neurotransmitters. Researchers have developed a variety of sensing strategies based on SWCNTs for the detection of neurotransmitters [40,86,145,146,147,148,149,150,151,152,153,154,155,156,157]. Regarding probe preparation and functionalization, Strano’s group [156] employed corona phase molecular recognition (CoPhMoRe) to identify the polymer phase adsorbed on the SWCNT surface, achieving specific dopamine detection with a linear response range from 10 nM to 10 μM and a detection limit of 11 nM. Landry’s group [157] obtained a serotonin probe through high-throughput screening of an ssDNA sequence library conjugated to SWCNTs. This probe exhibits a remarkable ~200% fluorescence enhancement and excellent selectivity, and remains response in brain slices. Jeong et al. [148] introduced a semi-automated platform for the reproducible fabrication of ssDNA-wrapped SWCNT dispersions, eliminating manual batch-to-batch variability and enables systematic parameter optimization to produce consistent nanosensors for the sensitive detection of serotonin. Williams and colleagues designed aptamer-functionalized SWCNT NIR fluorescent sensors for dopamine, demonstrating dynamic ranges between 30 and 200 nM and response times within 5 min in artificial cerebrospinal fluid [147]. Boghossian’s group [153,155] introduced locked nucleic acid (LNA) and xeno nucleic acid (XNA) to engineer SWCNT sensors with improved stability against cation-induced fluorescence changes, particularly from Ca^2+^, while retaining their capabilities for the label-free detection of dopamine. Kruss and colleagues [154] conjugated GFP-targeting nanobodies to the surface of DNA-wrapped SWCNTs, enabling targeted dopamine sensing at nanomolar levels. Bisker and colleagues [86] introduced oxygen defects into sodium cholate-dispersed (6,5) chirality single-walled carbon nanotubes (D-SWCNTs) and leveraged the fluorescence enhancement effect of dopamine on D-SWCNTs as well as the fluorescence quenching effect of serotonin on D-SWCNTs, thereby achieving fluorescent detection of these two neurotransmitters. The results showed that the detection limits of D-SWCNTs for dopamine and serotonin were 372 ± 156 nM and 113 ± 27 nM, respectively (Figure 5).

In terms of signal transduction methods, Kruss and colleagues [152] compared the reversible detection of neurotransmitters using electrical and optical sensing modes. Furthermore, they [151] demonstrated NIR fluorescence lifetime imaging microscopy based on SWCNTs for dopamine detection. Using the fluorescence lifetime of (GT)_10_-SWCNT as a metric, they achieved detection of dopamine in physiological buffer across a concentration range of 1 nM to 100 μM.

In the area of practical device integration, Landry and colleagues [150] developed a NIR catecholamine sensor based on SWCNT-functionalized optical fibers, which responded to dopamine across a concentration range of 10 nM to 1 μM and maintained response after 16 h of exposure to human blood plasma. Kruss and colleagues [149] further constructed a portable, low-cost levodopa detection reader based on a SWCNT sensor barcode, enabling the detection of 10 μM levodopa in serum.

### 4.3. Nucleic Acids

**Figure 6:**
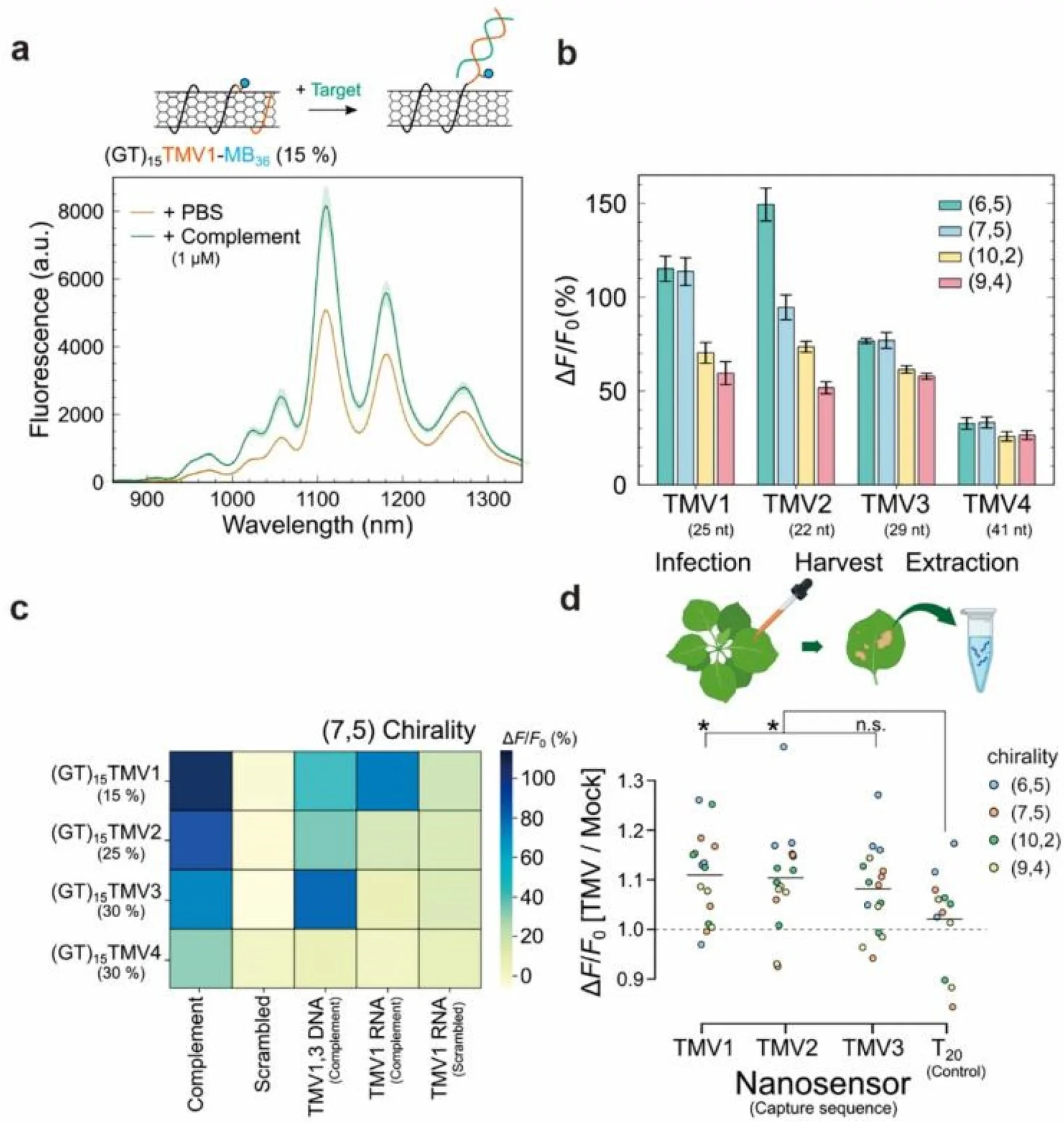
MB-mediated fluorescence energy transfer-based SWCNT nucleic acid detection. (**a**) NIR fluorescence spectra of (GT)_15_TMV1-SWCNT upon addition of 1 µM target ss DNA. (**b**,**c**) Chirality dependence of the integrated ΔF/F_0_ response (**b**) and corresponding heatmap (**c**) of SWCNT nanosensors. (**d**) Oligonucleotide detection of endogenous Tobacco Mosaic Virus in Nicotiana benthamiana leaf extracts. The asterisk (*) denotes the *P* value for statistical significance. Adapted with permission from Ref. [161], © National Academy of Sciences 2023. n.s. = no signification.

Functionalizing SWCNTs with ssDNA [158] enables the detection of nucleic acids through hybridization and modulation of the SWCNT fluorescence signal. Strano et al. [159] investigated the hybridization process of DNA and RNA on complementary ssDNA-NIR photoluminescence SWCNT corona phase complexes (CPs) for nucleic acid sensing using SARS-CoV-2 sequences as model analytes. The study found that the formation of secondary structures in the target is negatively correlated with hybridization efficiency. The best-performing complexes achieved detection limits of 11 nM and 13 nM for DNA and RNA targets, respectively. Landry et al. [160] utilized the ability of methylene blue (MB) to effectively quench the fluorescence of SWCNTs and designed a hybridization-induced displacement strategy of MB-labeled ssDNA from the SWCNT surface for oligonucleotide detection. The fabricated sensors achieved a tunable Δ*F*/*F*_0_ value of up to 150%. Based on this strategy, they further constructed four distinct nanosensors capable of selectively detecting RNA fragments of tobacco mosaic virus (TMV) and successfully distinguished TMV-infected plants from mock-treated control plants (Figure 6). Bisker’s group [161] developed fluorescent SWCNT sensors for highly specific and selective detection of microRNA biomarkers of acute myocardial infarction (AMI) via RNA-DNA hybridization. The selected sensor, SWCNT miDNA-208a, exhibited high specificity and selectivity toward the target sequence and demonstrated superior performance in serum.

### 4.4. Proteins

SWCNT-based NIR fluorescent nanosensors have achieved significant progress in the field of protein biomarker detection [162,163,164,165,166,167,168,169,170]. Heller et al. [165] utilized the NIR fluorescence properties of SWCNTs and employed bovine serum albumin (BSA) for surface passivation to develop a fluorescent nanosensor capable of quantitatively detecting the cancer biomarker urokinase-type plasminogen activator. Yudasaka et al. [163] modified the surface of NIR carbon nanotubes with IgG antibodies and combined them with magnetic beads to achieve immunoassay of IgG, with a detection limit of 600 pmol.

Strano et al. [164] cross-linked chitosan-wrapped SWCNTs into a gel, functionalized it with NTA-Ni^2+^, and conjugated it with a hexa-histidine-modified troponin T antibody. By monitoring changes in fluorescence intensity, they achieved detection of cTnT within 5 min, with a detection limit of 100 ng·mL^−1^ (2.5 nmol).

In terms of rapid response and high-sensitivity detection, Kruss et al. [170] constructed a CRP sensor using n-hexadecylphosphocholine-modified SWCNTs, achieving a 15% fluorescence response in human serum within 20 min and a detection limit below 13 mg/L. Landry et al. [167] developed an ACE2 non-covalently functionalized SWCNT sensor, which produced a 73% fluorescence turn-on response within 5 s upon surface immobilization upon exposure to SARS-CoV-2 spike protein. Strano et al. [166] further adsorbed PEG-phospholipid hybrid polymers onto SWCNTs to develop an antibody-free three-dimensional sensor that recognizes the SARS-CoV-2 N and S proteins within 5 min, with detection limits as low as 48 fM and 350 pM, respectively.

**Figure 7:**
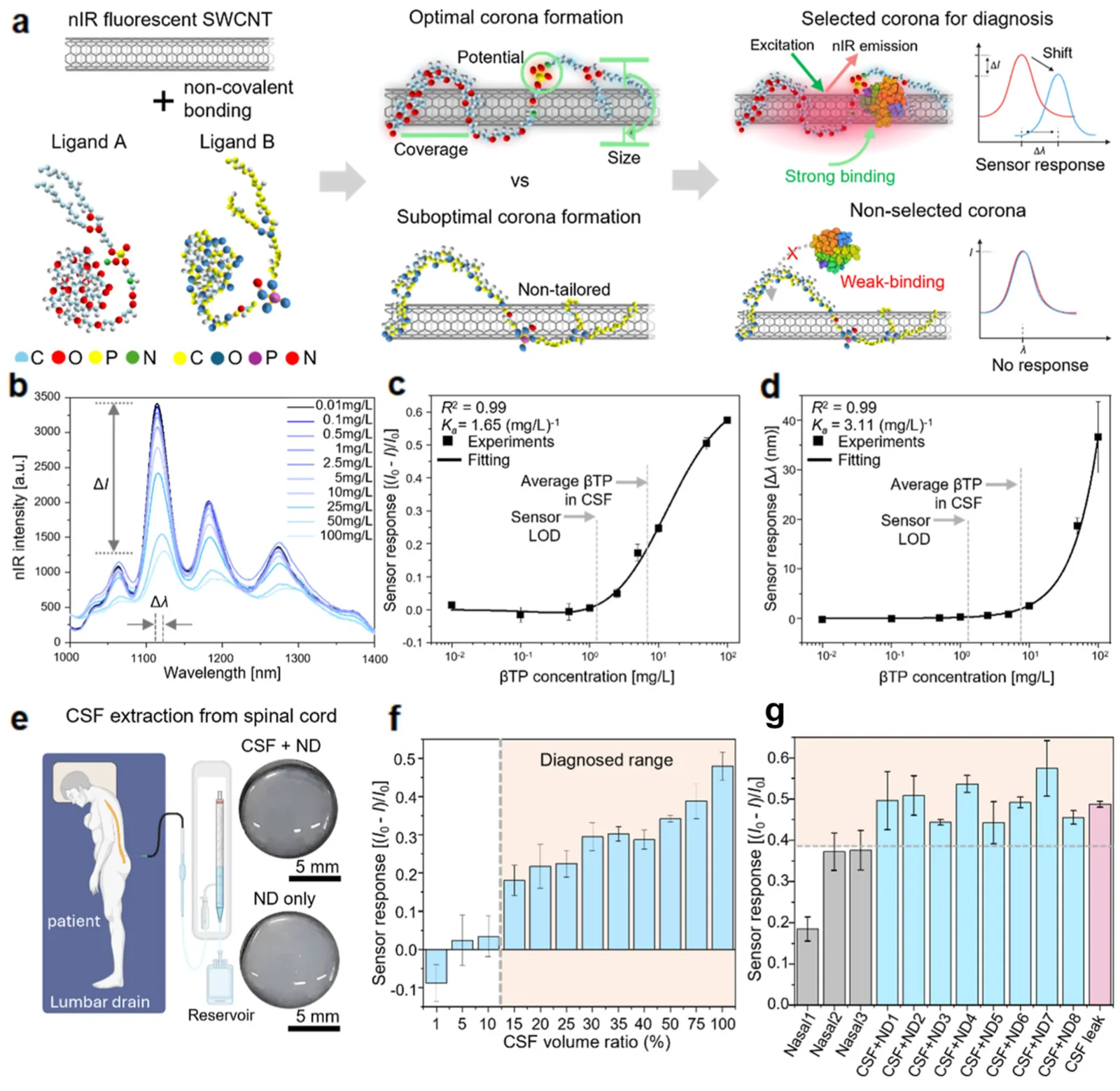
(**a**) Schematic illustration of the nanoparticle corona phase of nIR fluorescent SWCNTs for cerebrospinal fluid molecular detection. (**b**–**g**) Detection performance. (**b**) NIR fluorescence spectra of βTP at different concentrations and (**c**,**d**) calibration curves for intensity variation (**c**) and wavelength shift (**d**). (**e**) Left: schematic diagram of the lumbar drain method; right: photographs showing a comparison between simulated CSF leakage and nasal discharge alone. (**f**) Sensor response to simulated CSF leakage containing different ratios of PBS. (**f**) Sensor response to nasal alone, an equal-volume mixture of lumbar-drained CSF and nasal discharge, and a real CSF leak sample. Adapted with permission from Ref. [169], © American Chemical Society 2025.

Addressing the need for detection in complex clinical matrices, Cho et al. [169] employed 12 types of PEGylated lipids to non-covalently functionalize SWCNTs, achieving detection of β-trace protein as low as 1.46 mg/L while maintaining selectivity even in human nasal secretions (Figure 7). Williams et al. [168] screened poly-L-lysine as a non-covalent passivation agent, enabling a fluorescent antibody-conjugated SWCNT-based IL-6 nanosensor to achieve a clinically relevant detection range of 25–25,000 pg/mL in human serum.

### 4.5. Virus

**Figure 8:**
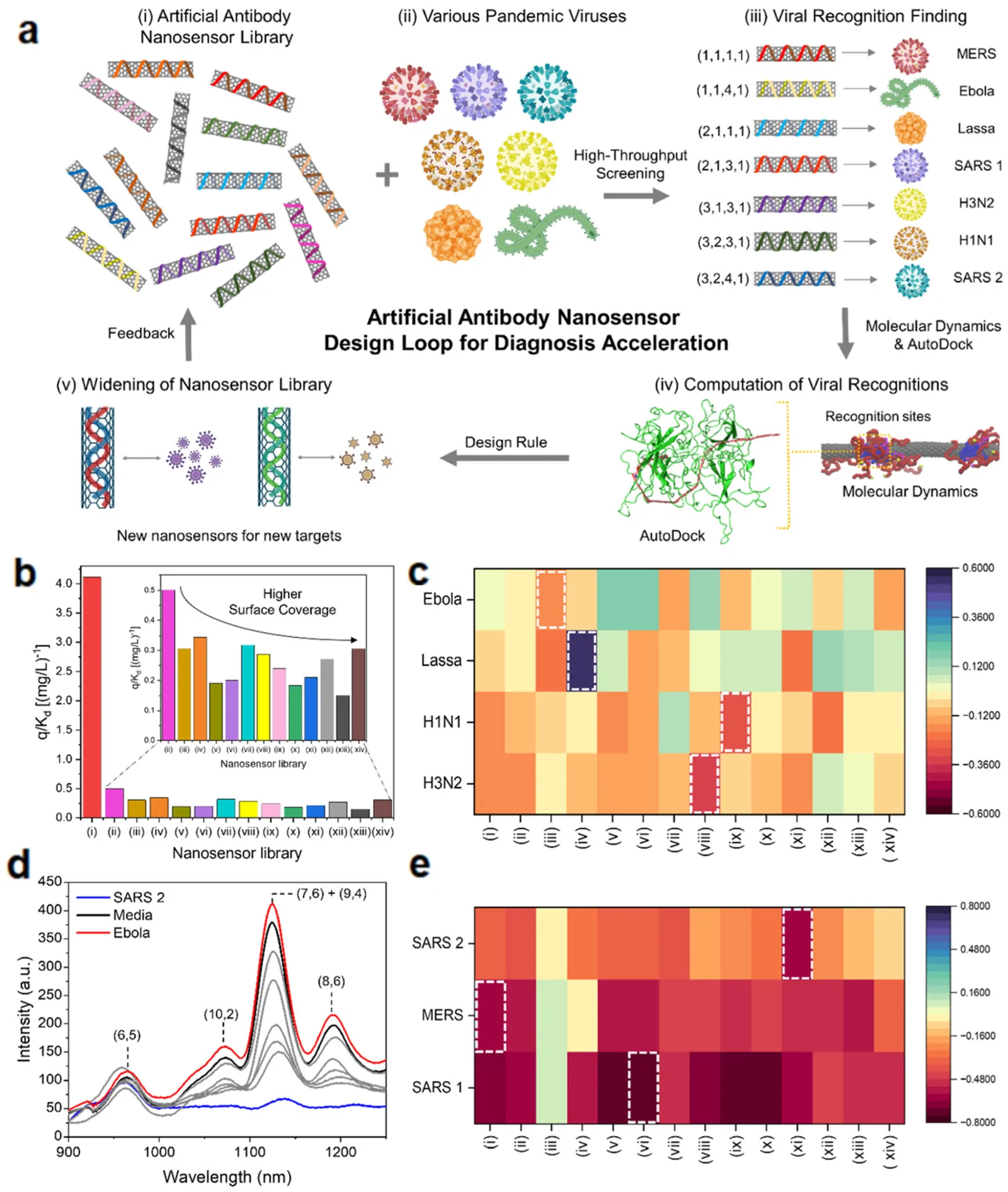
(**a**) Schematic diagram of the PEG-lipid corona phase of SWCNT-based nanosensors for universal virus recognition design. (**b**) Accessible surface area of PEG-lipid/SWCNT measured by molecular probe assay (MBA). (**c**,**e**) NIR fluorescence response heatmaps of the PEG-lipid/SWCNT nanosensor array for (**c**) Ebola, Lassa, H1N1 influenza, H3N2 influenza, (**e**) SARS-CoV-1, MERS, and SARS-CoV-2. (**d**) NIR fluorescence spectra of 18:1 PEG5000 PE/SWCNT for seven different viruses. Adapted with permission from Ref. [102], © American Chemical Society 2024.

SWCNTs functionalized with synthetic polymer corona have emerged as a versatile platform for label-free virus detection, enabling both direct sensor array construction and machine learning-enhanced spectral decoding. Cho et al. [102] used PEG-lipid (*n* = 14) functionalized SWCNTs to construct a sensor array for receptor-free virus recognition. The three-dimensional (3D) corona cross-interface of the nanosensor array enabled selective and sensitive detection of seven target viruses, including Middle East respiratory syndrome (MERS), Ebola, Lassa fever, severe acute respiratory syndrome coronavirus 1 (SARS-CoV-1), influenza H3N2, influenza H1N1, and severe acute respiratory syndrome coronavirus 2 (SARS-CoV-2), with detection limits of 3.4, 100.5, 16.6, 18.9, 13.3, 7.1, and 2.14 pM, respectively (Figure 8). Subsequently, this research group [171] developed a systematic machine learning framework to decode hidden spectral features within the NIR fluorescence of PEG-lipid-functionalized SWCNT nanosensors. Within this framework, the convolutional neural network (CNN) model achieved 99.68% accuracy even at concentrations 80-fold below the standard detection limit, and Shapley additive explanations (SHAP) analysis further revealed chirality-dependent features, particularly in the 1000–1086 nm region. This model not only predicted virus adsorption ratios in mixed samples but also distinguished unknown viruses (e.g., H1N1) with 100% accuracy at a 0.9 confidence threshold.

### 4.6. In Vivo Detection

NIR fluorescent sensors based on SWCNTs have demonstrated considerable potential for biological detection in plants and animals owing to their exceptional photostability, deep tissue penetration, and minimal interference from biological background signals. In recent years, many studies have achieved real-time, non-destructive monitoring of plant hormones, metal ions, and animal hormones through the development of functionalized SWCNT-based nanosensing platforms.

In the area of plant hormone detection, Jang et al. [172] developed an indole-3-acetic acid (IAA) nanosensor based on Corona Phase Molecular Recognition (CoPhMoRe). This sensor enables the detection of IAA in Arabidopsis thaliana and allows visualization of its distribution in living plant tissues. Furthermore, the sensor can detect plant responses to abiotic stresses such as shade and high temperature stress. Strano et al. [173] designed polymer-wrapped SWNTs to form unique corona phases capable of selectively binding bioactive gibberellins (GA_3_ and GA_4_), thereby constructing a nanosensor for gibberellins (GAs). In combination with a coupled Raman/NIR fluorimeter, this sensor achieved detection of cellular gibberellin levels in the roots of Arabidopsis thaliana, lettuce, and basil.

**Figure 9:**
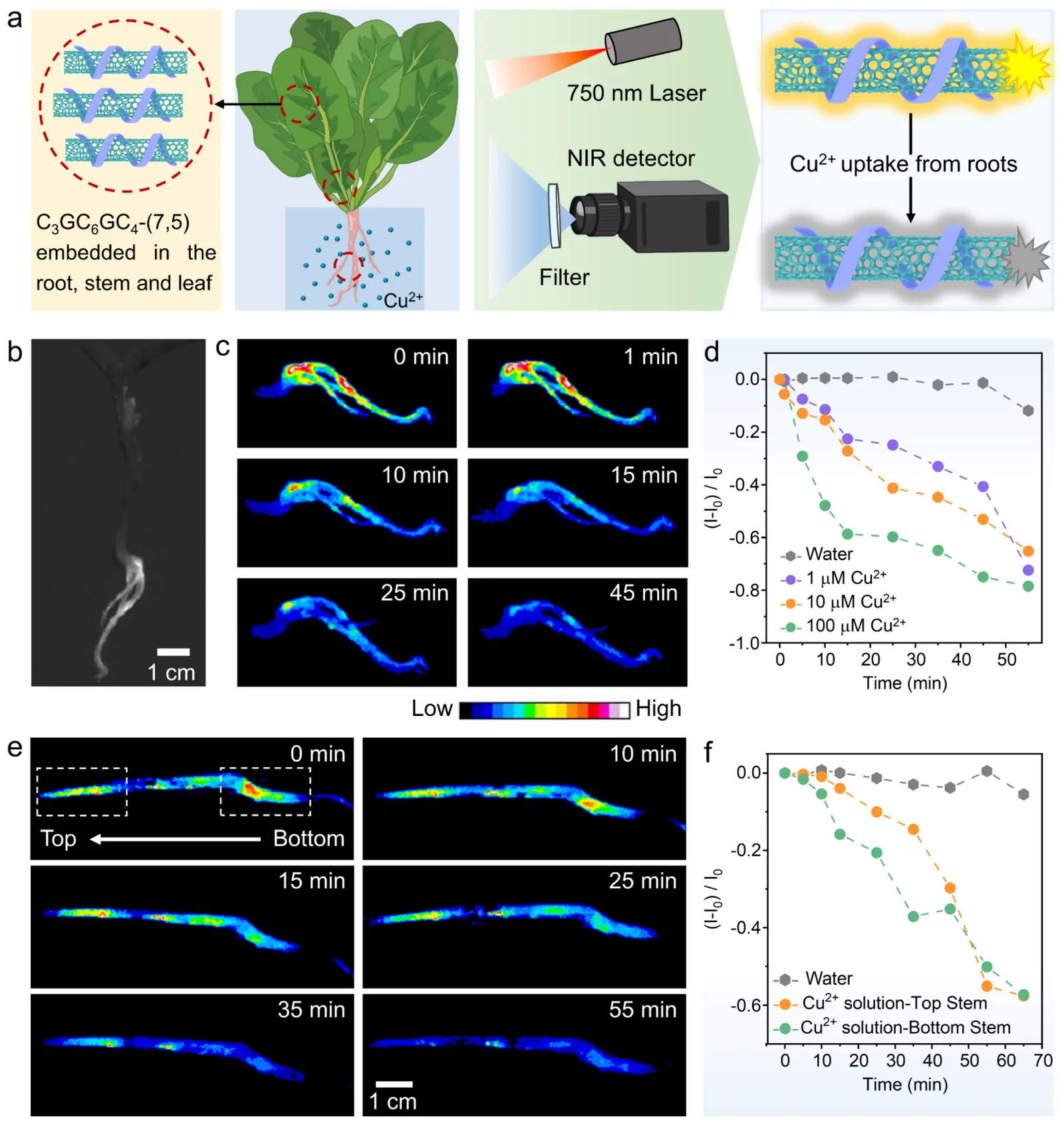
(**a**) Schematic illustration of a (7,5) SWCNT-based NIR nanosensor for real-time detection of Cu^2+^ in plants using an NIR camera and a 750 nm laser. (**b**) Bright-field image of a bean seedling treated with (7,5) SWCNT. (**c**) False-color images showing time-dependent fluorescence changes in the roots of a bean seedling cultured in a 10 μM Cu^2+^ solution. (**d**) Time-dependent fluorescence intensity curves in the roots of bean seedlings cultured in various concentrations of Cu^2+^ solutions. (**e**) False-color images showing time-dependent fluorescence changes in the stem of a bean seedling cultured in a 10 μM Cu^2+^ solution. (**f**) Time-dependent fluorescence intensity curves in the stem of bean seedlings cultured in Cu^2+^ solutions. Adapted with permission from Ref. [175], © American Chemical Society 2024.

In the field of plant metal ion detection, Strano et al. [174] developed NIR fluorescent nanosensors based on SWNTs using CoPhMoRe technology for iron detection and differentiation of its oxidation states (Fe(II) and Fe(III)), achieving a detection limit as low as 10 nM. Among these, an anionic poly(phenylene ethynylene) (PPE) polyelectrolyte-wrapped SWNT exhibited a fluorescence enhancement of up to 200% in response to Fe(II) and a fluorescence quenching of 85% in response to Fe(III), enabling spatiotemporal analysis of iron uptake processes in leaves and the root-to-shoot translocation pathway. Lin et al. [175] developed a copper ion detection platform using ordered DNA-wrapped, purified single-chirality carbon nanotubes (scCNTs). Among these, the C3GC6GC4-(7,5) complex achieved a detection limit for Cu^2+^ as low as 1.20 pM, while showing no cross-reactivity against 13 interfering ions. This sensor has been successfully applied to the real-time, rapid, and quantitative detection of Cu^2+^ uptake in living plants (Figure 9).

In the field of in vivo biosensing in animal models, Iverson et al. [176] demonstrated that PEGylated NIR fluorescent SWCNT sensors enabled selective detection of local nitric oxide (NO) with a detection limit of 1 µM via intravenous injection into mice. More recently, Landry et al. [177] synthesized a NIR oxytocin nanosensor (nIROXT) by leveraging the intrinsic tissue-transparent fluorescence of SWCNTs and the molecular recognition capability of an oxytocin receptor peptide fragment, enabling imaging of oxytocin in the brain. The results revealed that in the paraventricular nucleus of C57BL/6 mouse brain slices, oxytocin release sites had a median size of 3 μm, demonstrating the sensor’s capability to measure neuropeptide release in brain tissue.

## 5. Conclusions and Outlook

Significant progress has been made in the dispersion, chiral sorting, and optical sensing of SWCNTs. For dispersion, physical methods like ultrasonication offer a straightforward but often unstable approach, while chemical non-covalent strategies (polymer/DNA wrapping, surfactant adsorption, and π-π stacking) provide more robust and stable dispersions without damaging the nanotube’s intrinsic sp^2^ framework. In chiral sorting, ATPE and DGU offer ultrahigh purity and even enantiomer resolution but suffer from low throughput and limited scalability; GCC is facile for isolating multiple chiralities yet is hampered by batch-to-batch reproducibility issues; and polymer-based sorting enables convenient one-step enrichment but generally yields lower purity and is effective only for a restricted set of (*n*,*m*) species. In optical sensing, SWCNT-based NIR fluorescent sensors have demonstrated outstanding versatility across a wide range of analytes. They have achieved nanomolar to picomolar detection limits for toxic metal ions (Hg^2+^, Ag^+^, Cu^2+^), enabled sensitive detection of neurotransmitters (dopamine and serotonin), and facilitated nucleic acid and protein detection with rapid response and high selectivity. Furthermore, machine learning-assisted virus identification and real-time in vivo monitoring of plant hormones and neuropeptides highlight the broad applicability of these platforms in environmental, agricultural, and neurobiological research. Compared with organic fluorophores and quantum dots, SWCNTs possess unique merits for in vivo sensing, including stable NIR fluorescence, large Stokes shifts, excellent photostability, and deep tissue penetration. Nevertheless, they still face limitations such as batch-to-batch heterogeneity, bundling-caused fluorescence quenching, and unresolved biosafety risks. By contrast, organic dyes are readily functionalized and generally biocompatible, yet they are prone to photobleaching and lack deep tissue penetration. Quantum dots deliver high brightness and tunable emission wavelengths, though their heavy-metal composition introduces toxicity hazards.

Despite the promising proof-of-concept results, the field faces several unresolved critical challenges that hinder the standardized and practical translation of SWCNT sensing technology. First, scalable, low-cost, and high-purity production of monochiral SWCNTs remains a major bottleneck, as current separation and direct growth methods cannot simultaneously satisfy large-scale manufacturing and chiral uniformity requirements. Second, batch-to-batch variability in dispersion quality, sorting efficiency, and surface functionalization severely undermines experimental reproducibility and cross-laboratory result consistency. Third, functionalized SWCNT sensors suffer from insufficient long-term colloidal and optical stability, with ligand shedding and structural degradation causing persistent signal drift during prolonged detection. Fourth, accurate quantitative sensing in complex biological matrices remains challenging due to ubiquitous non-specific adsorption and background interference, restricting the reliability of in situ biological detection. Hence, anti-fouling coatings, robust surface anchoring, and standardized sample pretreatment are necessary to improve sensor reliability in complex biological environments. Furthermore, SWCNTs exhibit attractive merits for in vivo biosensing, including NIR fluorescence, high photostability and tunable surface functionalization. Nevertheless, systematic evaluation of their biocompatibility, in vivo biodistribution, and long-term biosafety remains insufficient, which creates barriers to both clinical and agricultural translation. Notably, there is a universal absence of unified evaluation criteria for sensing performance parameters, leading to inconsistent result reporting and hindering standardized technological iteration.

Future research should target solving the above bottlenecks to promote the industrialization and clinical translation of SWCNT multifunctional sensing platforms. The foremost challenge lies in scalable, high-yield production of well-defined single-chirality SWCNTs to replace polychiral mixtures, as their unique fluorescence fingerprints and predictable analyte interactions are indispensable for high sensitivity and reliable quantitative sensing. A second critical challenge is sensor performance degradation within complex biological matrices, which calls for standardized pretreatment, modification, testing and reporting protocols together with robust surface anchoring and anti-fouling coatings to improve stability, reproducibility and anti-interference capability; integrating chirality-resolved fluorescence fingerprints with machine learning can further enable advanced multiplexed and intelligent sensing. Third, inadequate knowledge of long-term biosafety remains a major barrier to in vivo and clinical translation, requiring comprehensive assessment frameworks for biocompatibility, biodistribution and biodegradability, alongside unified industry-wide sensing performance metrics to standardize experimental evaluation. With continued advances in material fabrication, surface functionalization and data analytics, standardized high-performance SWCNT NIR sensing platforms hold great promise for practical real-world deployment.

## Figures and Tables

**Table 1:** Comparison of non-covalent dispersion strategies for SWCNTs.

Method	Mechanism	Advantages	Limitations
Polymer wrapping	π–π stacking and steric hindrance	High selectivity, strong and stable dispersion, engineered aqueous solubility; scalable in principle	Solvent dependency, removal difficulty, sensing/functionalization interference, high cost, moderate dispersion efficiency, limited industrial scalability
DNA/ssDNA wrapping	π–π stacking, hydrogen bonding, electrostatic interactions	Sequence-dependent selectivity, excellent aqueous dispersibility, biocompatible, chirality-selective sorting, high sorting efficiency	Enzymatic susceptibility, poor thermal stability, pH/ionic-strength dependence, high cost, poor scalability, slow sorting process
Surfactant adsorption	Hydrophobic interactions, electrostatic/steric repulsion	Simplicity, low cost, aqueous dispersibility, charge-repulsion stabilization, high scalability, fast dispersion efficiency	Low chirality selectivity, surfactant removal difficulty, surface effects, potential cytotoxicity, moderate stability, low selectivity
Aromatic small molecules	π–π stacking	Non-covalent bonding, structure preservation, and group introduction, low cost	Poor dispersion, limited stability, time-dependent desorption, low-to-moderate dispersion efficiency, poor dispersion quality

**Table 2:** Comparison of four typical chiral SWCNT sorting methods.

Method	Separation Principle	Advantages	Limitations
Aqueous two-phase extraction (ATPE)	Partition coefficient difference in surfactant/ssDNA-wrapped SWCNTs between two immiscible aqueous phases	Mild conditions; capable of separating multiple (*n*,*m*) species and enantiomers; simple operation with multi-step extraction	Trade-off among chirality purity, yield and processing time; limited throughput
Density gradient ultracentrifugation (DGU)	Difference in buoyant density of surfactant/polymer-coated SWCNTs under ultrahigh centrifugal force	Ultrahigh separation precision; realizes enantiomer separation; applicable to armchair SWCNTs	Requires ultrahigh centrifugation (≥100,000 rpm); long processing time; low throughput
Gel column chromatography (GCC)	Difference in selective adsorption affinity between surfactant-capped SWCNTs and porous gel stationary phase	Widely used at lab scale; separates abundant chiral species and enantiomer pairs	Vulnerable to batch-to-batch reproducibility issues
Polymer-based wrapping sorting	Selective π–π wrapping of conjugated polymers toward specific (*n*,*m*) SWCNTs	Convenient operation; good scalability for certain chiralities	Restricted applicable chirality range; limited achievable purity

## Data Availability

No new data were created or analyzed in this study. Data sharing is not applicable to this article.
